# Dual Functionality of Chitosan in Chia Oil-in-Water Emulsions: From Interfacial Behavior to Short-Term Stability

**DOI:** 10.3390/foods15203741

**Published:** 2026-10-20

**Authors:** Fátima Vela, Francisco Carrillo, Victor M. Pizones Ruiz-Henestrosa, Cecilio Carrera, Nuria Calero

**Affiliations:** Department of Chemical Engineering, Higher Polytechnic School, University of Seville, C/Virgen de África, 7, E41011 Seville, Spain; fvela1@us.es (F.V.); cfuente@us.es (F.C.); vicpizrui@us.es (V.M.P.R.-H.); cecilio@us.es (C.C.)

**Keywords:** interfacial tension, interfacial rheology, egg yolk, chitosan, chia oil, food emulsion, emulsion stability

## Abstract

Physical stability of chitosan (CH)-based emulsions was investigated and compared with emulsions formulated with egg yolk (EY), a conventional food emulsifier. Additionally, a mixture of both components was examined to assess its potential to improve emulsion performance. Emulsion quality was characterized by evaluating interfacial properties, including interfacial tension and interfacial rheological properties, as well as the evolution of droplet size distribution using laser diffraction, optical microscopy, visual inspection, and multiple light scattering in chia-oil-in-water emulsions. Despite the higher interfacial tension reduction exhibited by egg yolk, emulsions stabilized solely by chitosan demonstrated significantly higher resistance to creaming and coalescence. In contrast, mixed CH/EY systems did not exhibit improved stability compared with CH-only emulsions. These observations indicate that emulsion stability was governed not only by interfacial properties but also by stabilization mechanisms operating in the continuous phase. Overall, the findings demonstrate that crayfish-derived chitosan can effectively function as a multifunctional ingredient in realistic multicomponent acidic food emulsions, providing both emulsifying and stabilizing capabilities and highlighting its potential for clean-label food applications.

## 1. Introduction

Oil-in-water (O/W) emulsions are widely used in food products such as dressings, sauces, beverages and functional foods. Their shelf life is largely determined by their physical stability, since emulsions are thermodynamically unstable systems that may undergo creaming, flocculation, coalescence, Ostwald ripening or phase separation during storage [1]. Therefore, the development of ingredients capable of promoting both emulsion formation and stabilization is of considerable technological interest. Conventional food emulsifiers, particularly proteins, are widely employed because they adsorb at the oil–water interface, reduce interfacial tension and form protective viscoelastic layers around dispersed droplets [1,2]. However, emulsion stability is not governed solely by interfacial activity, but also by factors such as adsorption kinetics, interfacial rheological properties and the physicochemical characteristics of the continuous phase [3]. Therefore, increasing attention has been devoted to biopolymers capable of simultaneously providing emulsifying and stabilizing functions. Among these, chitosan (CH) has been extensively investigated due to its ability to improve emulsion stability through electrostatic interactions and viscosity enhancement [3,4]. Nevertheless, most studies have focused on model systems and have mainly addressed its role as a stabilizing biopolymer, while limited information is available regarding its dual functionality as both an interfacial active and stabilizing ingredient under realistic food formulation conditions. Therefore, a knowledge gap remains concerning the relationship between the interfacial behaviour of chitosan and its contribution to emulsion stability in complex food systems.

In the 1970s, the invasive crayfish species *Procambarus clarkii* (red swamp crayfish) was introduced into the marshes of the Guadalquivir River. Its introduction into different ecosystems has had a significant impact on local aquatic communities, altering nutrient cycles, displacing native species and modifying the structure of aquatic habitats [5]. Despite the environmental problems caused by its invasion, the red crayfish also has interesting aspects from an economic and scientific point of view. In certain areas, this species has been targeted for commercial fishing because of its culinary appeal and the market demand for its meat. In fact, the Lower Guadalquivir marshes has become the leading producer and exporter of this crustacean [6]. Nevertheless, its fishing results in more than 2000 tons of exoskeleton waste annually when it is commercialized in peeled form [7]. In fact, the disposal of waste materials from shellfish, shrimp and fish is a serious problem in coastal areas around the world. Thus, the selective isolation of a bioactive material from these wastes, such as chitin, is an easy way to not only reduce environmental pollution due to the presence of these unused biomaterials but also to increase the production of bio-innovative products.

Chitin is the second most abundant natural polymer in nature, after cellulose [8]. Its chemical structure is similar to that of cellulose, with the difference that the former possesses an acetamide group instead of the C2 hydroxyl group of cellulose [9]. It is a high molecular weight linear polysaccharide composed of repetitive units of N-acetyl-D-glucosamine (GlcNAc) that are linked by β-(1,4) glycosidic bonds [10]. Its chemical structure includes functional groups such as hydroxyl groups (-OH) and amino groups (−NH_2_). The chemical modification of chitin is carried out through the partial or complete deacetylation of the acetyl groups (-COCH_3_) and chitin is considered converted into chitosan when at least 50% of its acetyl groups have been eliminated [11]. Chitosan is soluble in acid solutions and possesses different chemical and physical properties than chitin, with greater reactivity and solubility [12]. This chemical modification of chitin allows the generation of a wide variety of derivatives (different degrees of deacetylation and molecular weights) with different specific properties [13]. Properties such as their notable thermal stability, hygroscopic capacity, biodegradability or low toxicity give chitosan particular appeal for various applications including biomedical technology, cosmetics, agriculture, food, water treatment, textile and paper industries [14]. In addition, chitosan contributes functionality or functional properties to the different systems, specifically it acts as an antimicrobial agent, anti-grease agent, emulsifier, and moisturizer, making them more competitive and with higher added value. In the 1980s, the concept of functional foods emerged, referring to foods that, in addition to their nutritional value, contain biologically active components. These components provide beneficial health effects and reduce the risk of contracting certain diseases [15]. This is achieved thanks to functional ingredients such as chitosan, whose characteristics define the properties of the food in which they are integrated [16].

An emulsion is a system widely used in many applications, which is based on a thermodynamically unstable dispersion of two or more immiscible or partially miscible liquids. Emulsions are typically composed of water (polar phase) and oil (nonpolar phase), one of which is dispersed as small spherical droplets in the other. Despite thermodynamic instability, kinetically stable emulsions can be formed for periods suitable for practical applications by adding emulsifiers (amphiphilic substances) and/or stabilizers (polysaccharides), which improve emulsion stability in different ways [17].

Both egg yolk (EY) and egg white contain a mixture of protein and non-protein components that are surface-active. Egg ingredients can be commercialized in different forms for their use in food emulsions, including fresh egg yolks, frozen egg yolks, dried egg yolks, fresh whole eggs, frozen whole eggs, and dried whole eggs. In the food industry, egg white is mainly used to stabilize foams, while egg yolk is most commonly used to stabilize emulsions [18]. The egg yolk is divided into two fractions, one part called plasma (aqueous phase) and granules (0.3 μm–2 mm insoluble denser structures), which contain approximately 52% dry matter (in which 65% fat, 31% protein and 4% carbohydrates, vitamins and minerals) [19]. Egg yolk proteins act as emulsifiers and are primarily livetins and low-density lipoproteins (LDL). The lipid in LDL consists of 70% neutral lipid, 26% phospholipids (71–76%, phosphatidylcholine, 16–20% phosphatidylethanolamine, and 8–9% sphingomyelin and lysophospholipids), and 4% free cholesterol [20].

Chitosan, being a polysaccharide, primarily acts as a stabilizer in an emulsion by increasing the viscosity of the continuous phase and by its ability to form a three-dimensional gel-like network in the continuous medium [21]. However, chitosan is usually not classified among the group of polysaccharides with good emulsification properties, but several studies have shown that chitosan can in fact act as the sole emulsifier and produce emulsions of acceptable stability [22,23,24,25,26,27]. It has also been reported that it exhibits surface activity and it behaves as a surface-active hydrophilic Pickering agent [28,29]. These surface-active agents provide a physical barrier against droplet coalescence and improve the long-term stability of emulsions by reducing the interfacial energy between the immiscible phases through the adsorption of solid particles at the interface (formation of a steric barrier).

Chia oil is obtained through cold pressing of chia seed, and it represents a rich natural plant-based source of omega-3, with a content of about 65% of this component. The type of omega-3 found in chia oil is alpha-linolenic acid (ALA), which is a crucial dietary source and its inclusion in the diet is essential for health since, among other benefits, it can help correct dyslipidemia, that is, reducing LDL cholesterol and triglycerides while increasing HDL cholesterol. It also contains quantities of bioactive substances, such as antioxidants, tocopherols and phytosterols, which make it a highly valuable product [30].

Although numerous studies have investigated chitosan and egg-yolk-based emulsion systems [31,32,33,34], research addressing the combined use of both ingredients remains limited. Previous studies have mainly focused on emulsions prepared with egg yolk hydrolysates or on Pickering emulsion gels stabilized by egg-yolk–granule/chitosan complexes [35,36]. Consequently, the combined behaviour of chitosan and egg yolk in conventional food emulsions remains largely unexplored, particularly in realistic multicomponent food formulations.

Therefore, this work aimed to investigate the mechanisms governing the stability of complex acidic food emulsions containing crayfish-derived chitosan and egg yolk within a realistic multi-component food formulation. Rather than examining chitosan solely as a thickener, this study examines how the combined presence of chitosan and egg yolk influences interfacial and bulk emulsion properties under acidic conditions.

## 2. Materials and Methods

### 2.1. Materials

Chitosan (CH) with a deacetylation degree of 85.03%, a molecular weight of 262.5 kDa, and a purity of >98% (ash content < 1.5%) was isolated from crayfish biowastes as previously described [28]. Commercial egg yolk (EY) with 10 wt.% added NaCl and a dry matter content of 52 wt.% was provided by Eurovo (Valladolid, Spain).

White wine vinegar (acidity of 6%) from the Hacendado brand (Murcia, Spain), salt provided by the IFA brand (Madrid, Spain); Potassium sorbate supplied by PanReac (Barcelona, Spain); Lemon juice preserved in an environment at −10 °C provided by IFA (Toledo, Spain).

Organic Chia oil was supplied by Bidah Chaurel (Murcia, Spain) and was used as the oil phase without further purification to check the performance of the adsorbed components in a real interface.

Acetic Acid glacial (CH_3_COOH), Hydrochloric acid (HCl), ortho-Phosphoric Acid 85% (H_3_PO_4_), Sodium Acetate Trihydrate (CH_3_COONa⋅3H_2_O) and Sodium Hydroxide (NaOH) were purchased from Panreac Química, S.A. (Castellar del Vallès, Spain). Acetyl-glucosamine (GlcNAc), D-glucosamine hydrochloride (GlcN) and Sodium Azide (NaN_3_) employed were from Sigma Aldrich (Steinheim, Germany).

All the reagents were of analytical grade and used without purification.

### 2.2. Methods

Aqueous continuous phase preparation:

The aqueous continuous phase was formulated to represent 90 wt.% of the O/W emulsion (10:90 oil-to-water mass ratio). This phase consisted of Milli-Q water, white wine vinegar, and lemon juice, providing an acidic pH of 3.0–3.5 required to fully dissolve CH. Potassium sorbate and sodium chloride were added as preservatives. Table 1 reports the composition of the aqueous phase in mass percentages (wt.%). Consequently, the final CH concentrations in the whole emulsion were 0.36, 0.45 and 0.54 wt.% (corresponding to 0.4, 0.5 and 0.6 wt.% in the aqueous phase), while EY represented 0.9 wt.% of the total emulsion (1.0 wt.% in the aqueous phase).

The solution containing Milli-Q water, vinegar, and dry CH polymer was kept under magnetic stirring for 24 h to guarantee complete polymer dissolution. Subsequently, the remaining ingredients were added, and the mixture was magnetically stirred until full homogenization.

#### 2.2.1. Interfacial Characterization

It should be noted that the interfacial characterization and emulsion studies were designed as two complementary but independent approaches. Interfacial experiments were performed under simplified and controlled conditions to investigate the adsorption behaviour and interfacial activity of chitosan and egg yolk at the oil–water interface. For this purpose, each component was evaluated individually at its saturation concentration and, additionally, as a 50:50 mixture to facilitate the interpretation of interfacial phenomena. In contrast, emulsion studies were conducted using concentrations and compositions closer to those employed in real food formulations, where the objective was to evaluate the overall performance of the ingredients within a complex multiphase system.

##### Interfacial Tension at Equilibrium

The interfacial tension (σ_eq_) was measured using a Sigma 701 tensiometer (KSV, Helsinki, Finland) using the Wilhemy plate method, with a roughened platinum plate. Prior to each assay, the platinum plate was cleaned by thoroughly washing with deionized water and isopropanol, and flame-sterilized using a Bunsen burner to eliminate organic contaminants. The different solutions were prepared and they were stored during 24 h at 4 °C before measurements. For the measurements at the O/W interface, the platinum plate was first immersed in and pre-wetted with the aqueous phase to ensure complete wetting, after which a 6–7 mm layer of oil was placed so that the platinum plate was completely covered [37].

The concentration of the aqueous phase solutions containing CH ranged between 0.0125 and 0.5% wt. Systems containing EY alone, as well as CH/EY mixtures at a 50:50 ratio, were also evaluated.

The evolution of interfacial tension (σ) with time was measured. Equilibrium (σeq) was assumed when σ did not change by more than 0.1 mN/m in 30 min. The σ_eq_ values were reproducible within ±0.5 mN/m. The experiments were repeated at least twice, and the $\sigma_{eq}$ values at different concentrations were used to determine the equilibrium interfacial tension profiles for each system studied.

##### Interface Shear Rheological Properties

The evaluation of the interfacial shear rheology was carried out using a Discovery HR-3 stress controlled rheometer (TA Instruments, New Castle, DE, USA) equipped with a Double Wall Ring (DWR) geometry [38]. The Pt-Ir ring has a cross-sectional wire radius of 0.48 mm and mean ring radius of 34,5 mm, while the Teflon trough features inner and outer radius of 31.0 mm and 39.5 mm, respectively (gap width 8.5 mm) [39].

To establish the interface, 18 mL of the continuous aqueous phase was loaded into the trough, and the ring was lowered until its median plane coincided with the surface, as verified by normal force detection. Subsequently, 12 mL of chia oil was carefully layered on top.

Mechanical spectra were obtained by means of frequency tests, which were carried out within the linear viscoelastic range (assessed through stress sweep tests that were performed). The frequency tests were performed from 0.01 to 0.3 Hz during CH adsorption until reaching the equilibrium (at 0, 15, 30, 45, 60 and 180 min). Time sweep tests were carried out over the protein adsorption (3 h) at 0.1 Hz. All experiments were carried out at 20.0 ± 0.1 °C.

##### Preparation of O/W Emulsions

A low energy method based on a rotor-stator system (L5M emulsifier, Silverson, Chesham, UK) was employed to prepare the emulsions at 10,000 rpm for 2 min. When preparing the O/W emulsions, only half of the aqueous continuous phase was emulsified with all the chia oil (selected dispersed phase). After that, the other half of the continuous phase was incorporated and magnetically stirred until homogenized properly to avoid damaging the CH network structure during emulsification. Different concentrations of CH higher than the saturation concentration were used. An emulsion formulated with EY alone was also prepared, as well as their mixture containing the same CH concentrations. The compositions of different emulsions prepared are shown in Table 1.

##### O/W Emulsions Characterization

Different experimental techniques were used to analyze the stability of the O/W emulsions formulated. In this sense, the measurements were carried out on the first day, once the emulsion was formed (day 0), and on the following day (day 1).

1. Droplet Size Distribution

The oil droplet size distribution (DSD) of emulsions was determined by static light scattering using a Mastersizer 2000 (Malvern Instruments, Malvern, United Kingdom). Measurements were performed using the Mie scattering theory, with a refractive index of 1.481 and an absorption index of 0.001 for the chia oil droplets, and a refractive index of 1.330 for distilled water, which was used as the continuous dispersant medium. Prior to analysis, emulsion samples were gently inverted three times and added dropwise to the measurement cell until an obscuration level between 7% and 9% was achieved. The pump and stirring speeds were set to positions 8 and 5, respectively, to maintain continuous sample circulation without inducing droplet disruption or air bubble entrapment.

The data obtained were evaluated in terms of the Sauter mean diameter (D[3,2]), the volume-weighted mean diameter (D[4,3]), and the Span parameter as an indicator of polydispersity.

(1) $$D_{3,2}=\frac{\sum_{i=1}^{N}n_{i}d_{i}^{3}}{\sum_{i=1}^{N}n_{i}d_{i}^{2}}$$

(2) $$D_{4,3}=\frac{\sum_{i=1}^{N}n_{i}d_{i}^{4}}{\sum_{i=1}^{N}n_{i}d_{i}^{3}}$$

where N stands for the total droplet count, d_i_ indicates the diameter of an individual droplet, and ni refers to the quantity of droplets having a diameter of d_i_.

(3) $$Span=\frac{\left(D\left[v,0.9\right]-D\left[v,0.1\right]\right)}{D[v,0.5]}$$

where D[v, 0.9] and D[v, 0.1] represent the 90th and 10th percentiles, respectively, while D[v, 0.5] denotes the median value. Each measurement was performed in triplicate at ambient temperature.

2. Microstructural Evaluation

The microstructure of emulsions was analyzed at room temperature using an optical microscope Axio Scope A1 (Carl Zeiss, Oberkochen, Germany) equipped with an AxioCam camera. Microphotographs were taken with a 20× objective. To improve the view of the flocs, all samples were diluted to 1:20 in distilled water.

3. Phase Separation Observation

Digital images of the emulsions placed into vials were taken to evaluate visually a possible phase separation at a macroscopic level. The different vials were stored at 25 °C.

4. Multiple Light Scattering

Global physical stability of emulsions was monitored with a vertical scan analyzer Turbiscan Lab Expert (Formulaction Company, L’Union, France), which is a non-invasive experimental technique. This equipment allows the turbidity profile of an emulsion along the height of a flat-bottomed glass tube to be scanned. Backscattering percentage (%BS) obtained as a function of the sample height was evaluated at room temperature for 24 h in order to determine the predominant mechanism of destabilization in each case as well as the kinetics of the destabilization process. The evaluation of these profiles, that represent a macroscopic fingerprint of the emulsions, leads to quantitative data related to the stability of the emulsions and allows comparison between the emulsions [40].

The results are reported as the average of two samples.

### 2.3. Data Processing and Statistical Analysis

Data processing, profile fitting, and graphical representations were performed using Origin 9.0 (OriginLab Corporation, Northampton, MA, USA). All experimental measurements were conducted in triplicate (*n* = 3), and results are reported as mean ± standard deviation. Statistical evaluation was carried out using RStudio software 4.6. (RStudio Team, Boston, MA, USA). One-way Analysis of Variance (ANOVA) followed by Tukey’s Honestly Significant Difference (HSD) post-hoc test was applied to evaluate differences among treatments at a significance level of *p* < 0.05. Independent statistical tests were performed for single (CH) and mixed (CH/EY) emulsion systems.

## 3. Results and Discussion

### 3.1. Interfacial Properties

#### 3.1.1. Interfacial Tension Measurement

**Figure 1:**
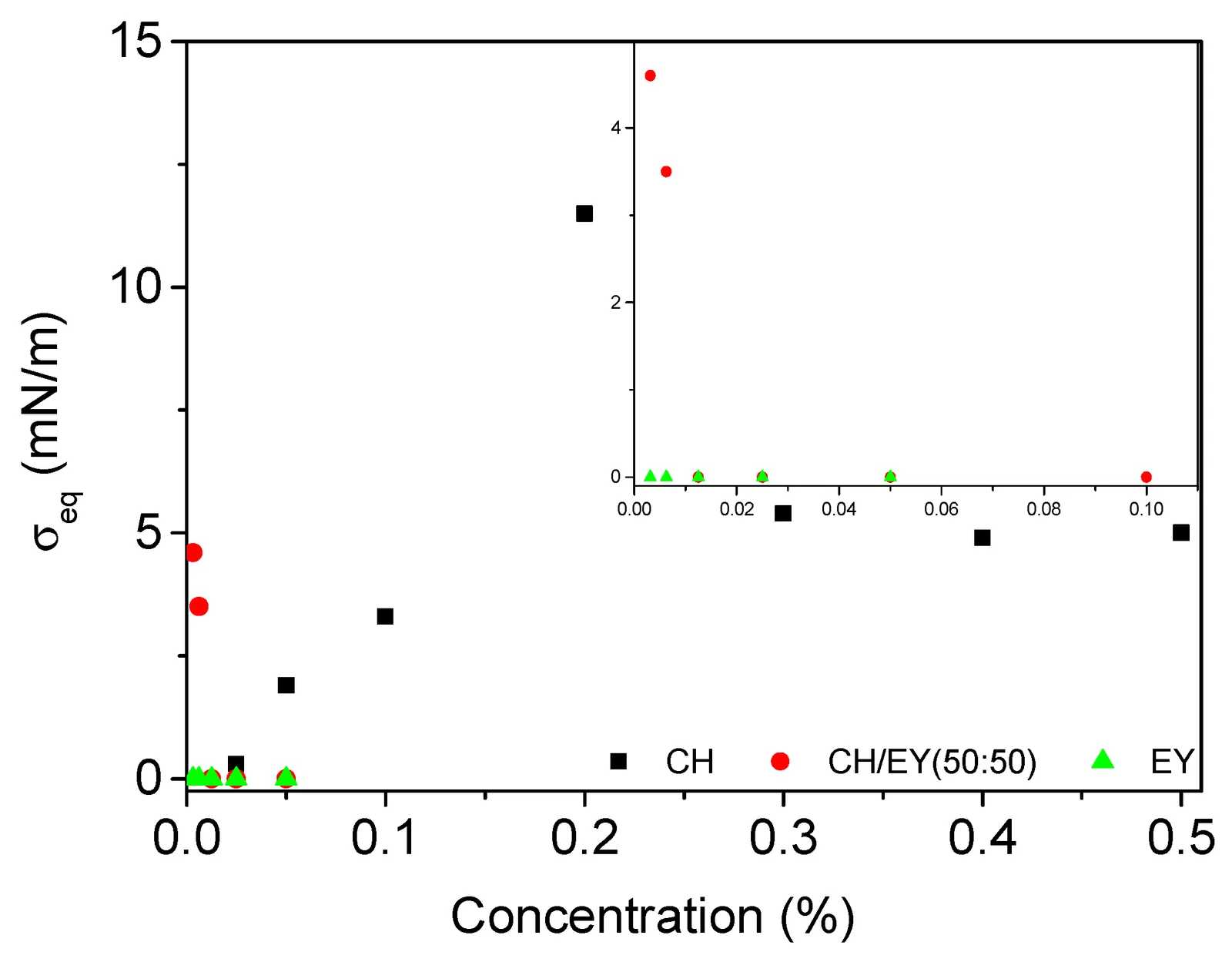
Experimental isotherm for equilibrium interfacial tension for CH (0.025–0.5% wt.); EY (0.003–0.05% wt.) and CH/EY mixture (50:50) (0.03–0.1% wt.) at the oil–water interface, measured by the Wilhelmy plate method at 20 °C. The inner box is an expansion of the data from EY and CH/EY.

Figure 1 shows the equilibrium interfacial tension profiles determined at the oil–water interface as a function of emulsifier concentration (ranging from 0.025 to 0.5% wt. for CH, and from 0.003 to 0.5% wt. for EY and the 50:50 mixture). The results indicate a general trend in which increasing emulsifier concentration leads to a progressive decrease in interfacial tension until a constant value is reached. The adsorption efficiency (AE) (saturation concentration) was found to be approximately 0.3% (wt.) for CH and 0.0125% (wt.) for the mixture, while EY exhibited the highest efficiency, with an AE below 0.00313% (wt.).

The observed evolution of the isotherms is directly linked to the increasing density of emulsifier molecules adsorbed and reorganized at the oil–water interface, which drives the reduction in interfacial tension [41]. Once the interface is saturated, the system reaches a minimum interfacial tension, and further increases in bulk concentration do not result in significant changes [42]. This behaviour suggests that, above the AE, additional molecules likely present as aggregates or micelles in the aqueous phase orient their hydrophobic moieties towards the interior to minimize contact with the solvent [43]. Although EY behaves as the most efficient emulsifier in terms of AE, these results indicate that EY proteins dominate the interfacial stabilization.

With respect to CH, it appears to act as a surface-active hydrophilic Pickering agent, leading to a decrease in interfacial tension as a consequence of particle adsorption at the interface [28]. At the lowest CH concentrations, interfacial tension values were lower than the interfacial activity (minimum interfacial tension at the plateau) (~5 mN/m). This behaviour can be attributed to the fact that the aqueous phase employed corresponds to a model food formulation containing vinegar, salt, lemon juice and potassium sorbate rather than a pure system. Consequently, surface-active impurities present in these ingredients may contribute to the pronounced reduction in interfacial tension when the CH concentration is insufficient to fully control the interface.

It is well established that impurities can significantly affect surface and interfacial tension, as increasing impurity concentration generally leads to a decrease in equilibrium interfacial tension [44,45]. In systems where intermolecular interactions between surfactant molecules are stronger than those involving other surface-active species, impurities may be displaced from the interface through competitive adsorption [46]. Similar minima in surface tension curves due to the presence of highly surface-active impurities have been reported by Esam. A. et al. [45], Chen et al., [47] and Tofani et al., [48]. Additionally, the presence of minor polar constituents in unrefined chia oil such as free fatty acids, mono- and diacylglycerols, phospholipids, and phytosterol should be considered as these surface-active species may readily absorb at the cold-pressed oil–water interface [49,50]. The use of unpurified chia oil in this study therefore contributed to the relatively low interfacial tension values observed (approximately 10 mN/m).

Regarding EY, its components, including low-density lipoproteins, phospholipids, livetins and cholesterol, exhibit a strong tendency to adsorb at the oil–water interface [51]. Among these, proteins are generally considered the primary contributors to interfacial adsorption [52]. Furthermore, Li et al. [53] reported that the interfacial properties of EY are correlated with its zeta potential and particle size, which are sensitive to both NaCl concentration and pH. These factors likely contribute to the extremely low, and in some cases non-measurable, interfacial tension values observed for EY and for the CH/EY mixed system. In the mixed system, the similarity of interfacial tension values to those obtained for EY alone suggests that interfacial adsorption is largely governed by EY components, in agreement with previous observations [54]. The 50:50 CH/EY mass ratio was intentionally selected as an idealized model system to compare the interfacial behaviour of the two surface-active species under equal mass availability.

#### 3.1.2. Interfacial Shear Rheological Properties

**Figure 2:**
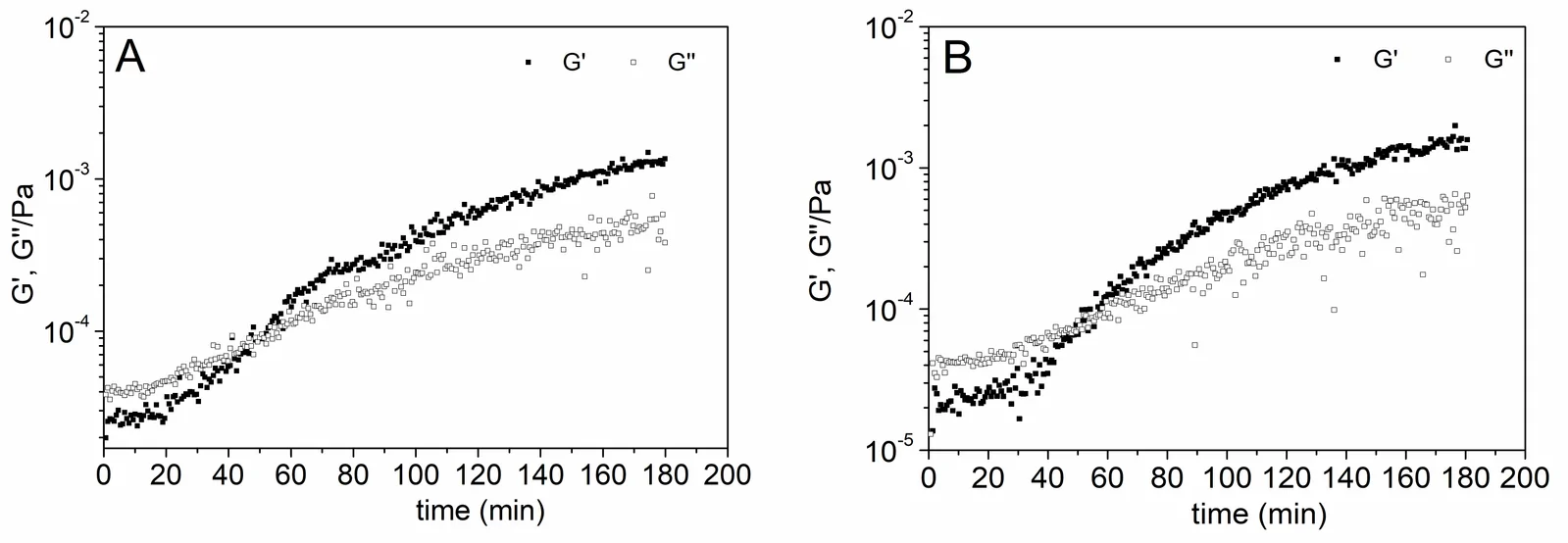
Evolution of interfacial shear viscoelastic parameters (G′ and G″) with adsorption time at 0.1 Hz and 0.12% strain amplitude, 20 °C for (**A**) an aqueous solution of EY (0.003% wt.) and (**B**) an aqueous solution of a mixture of CH and EY (0.0125%) at the saturation concentration level.

**Figure 3:**
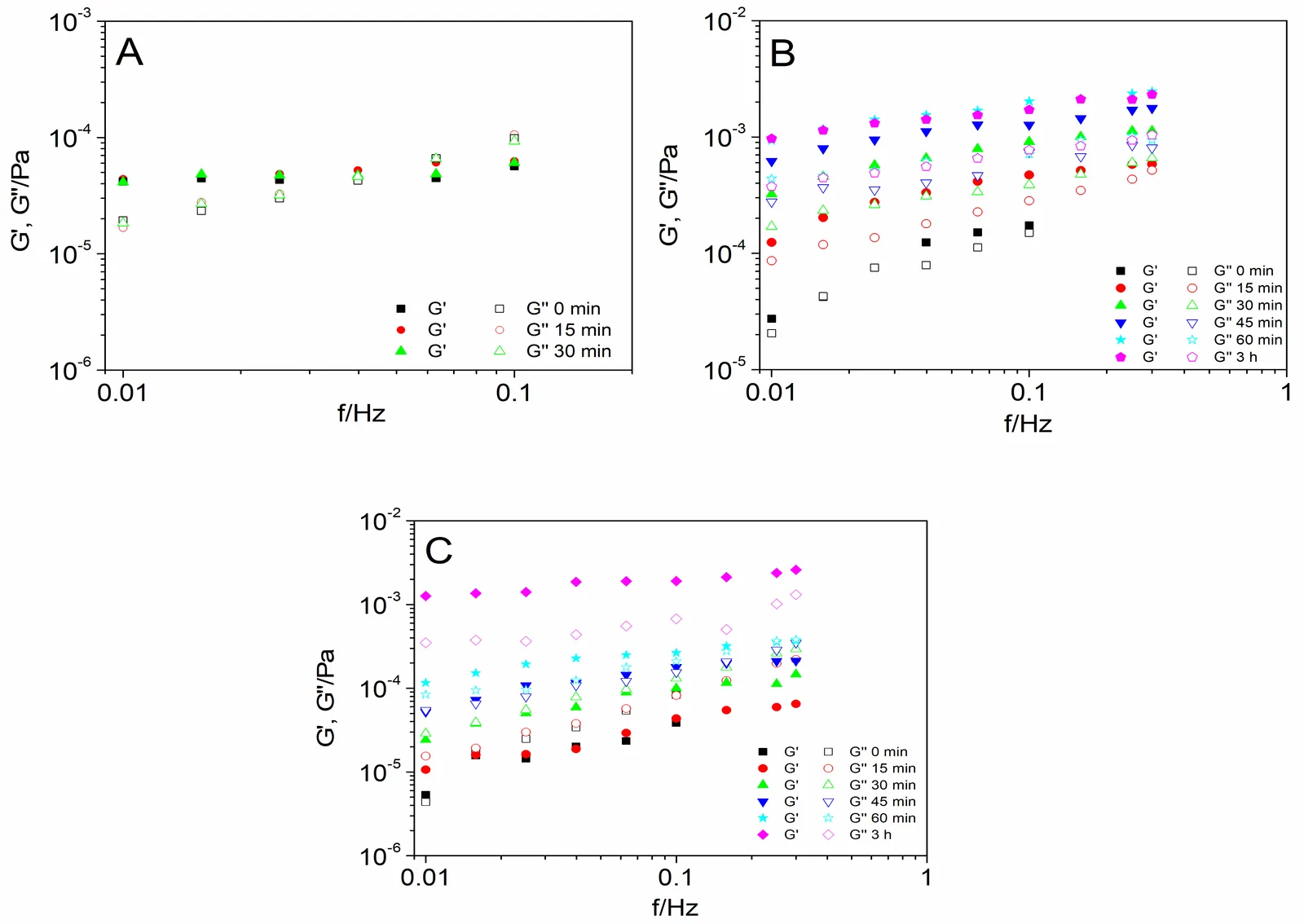
Interfacial oscillatory shear mechanical spectra (frequency sweeps, 0.01–0.3 Hz, 0.12% strain amplitude, 20 °C) obtained at increasing adsorption times (0, 15, 30, 45, 60 min and 3 h) during pseudo-equilibrium formation for: (**A**) an aqueous solution of CH (0.3% wt.); (**B**) an aqueous solution of EY (0.003% wt.); and (**C**) an aqueous solution of the mixture CH/EY (0.0125% wt.) at the saturation concentration level.

The interfacial viscoelastic properties were evaluated by linear interfacial shear rheology (Figure 2 and Figure 3), which provides information on the adsorption and the and structural organization of surface-active molecules at the interface [55].

First, the elastic modulus (G′) and viscous modulus (G″) of the interfacial films were measured as a function of time to investigate the adsorption process, ageing and structural reorganization of surface-active agent molecules (Figure 2). The results obtained for the CH system revealed that it did not show changes in G′ and G″ with time, which indicates that the pseudo-equilibrium was reached immediately once the interfacial film was created [28,56].

However, the results shown in Figure 2A,B indicated a different behavior for EY and mixed systems at the saturation concentration, respectively. In both cases, the adsorption of surface-active agent molecules on the O/W interface implied a rapid increase in the elastic and viscous interfacial moduli (G′s and G″s) with a tendency to reach a plateau value, indicative of pseudo-equilibrium [57]. At early adsorption times, G” was higher than G′ until a crossover point was reached at approximately 2500 s. At longer times, the elastic modulus became progressively higher than the viscous modulus, indicating the increase in elastic character as a consequence of the occurrence of strong interactions on the interface, or the formation of a densely compressed film. Similar behaviour has been reported for other protein-based interfacial systems [38,58,59]. The observation that both EY and CH/EY systems exhibit comparable trends suggests that interfacial adsorption is mainly controlled by EY components, which is consistent with the interfacial tension results presented in Figure 1.

Linear viscoelastic conditions were ensured by applying small interfacial deformations so that the interfacial structure remained unaltered (constant strain amplitude of 0.12%, within the linear viscoelastic region, LVR) [1] [38]. Although a crossover between G′ (ω) and G” (ω) was observed (Figure 3), the absence of a significant separation between both moduli indicates that the interfacial film cannot be classified as a strong gel. Instead, it behaves as a weak viscoelastic network with limited elastic dominance over the frequency range studied [60]. At higher frequencies, the response approaches a glassy regime, where the limited time available for configurational rearrangements of molecular backbones leads to behaviour similar to that observed in dilute systems.

Furthermore, no significant changes in the mechanical spectra with adsorption time were detected, indicating that the interfacial film was formed almost instantaneously [28].

Figure 3B and Figure 3C show the evolution of the interface oscillatory shear mechanical spectra obtained during the adsorption of the surface-active agents until pseudo-equilibrium was reached for EY and CH/EY mixture, respectively. The evolution of the interfacial structure was analysed by frequency sweeps (0.01–0.3 Hz) at different times (t = 0, 15, 30, 45, 60 min and 3 h) using a constant strain amplitude of 0.12% (within the LVR) for EY (Figure 3B) and mixed system (Figure 3C). Both systems exhibited a gel-like mechanical behaviour corresponding to the plateau region, characterized by storage moduli (G′s) higher than loss moduli (G”s) across the full frequency range studied [38].

For both systems, G′ and G” increased with adsorption time. At short times, the EY system exhibited a predominance of the viscous response, indicating a weakly structured interfacial film in formation. With increasing adsorption time, both moduli increased until G′ exceeded G”, reflecting the development of a fully formed interfacial film. In contrast, at early adsorption times the mixed system presented a mechanical spectrum similar to that of CH, likely due to the faster, initial adsorption of CH molecules. At adsorption times longer than 15 min, G′ exceeded G” throughout the frequency range and both moduli continued to increase until pseudo-equilibrium was reached after approximately three hours. At pseudo-equilibrium, EY and CH/EY systems showed very similar mechanical spectra, confirming the dominant role of EY molecules in controlling the interfacial layer.

### 3.2. Emulsifying Capacity Studies

To evaluate the emulsifying capacity of CH, O/W emulsions were prepared at food-relevant concentrations using CH at 0.4, 0.5, and 0.6 wt.%, either alone or in combination with 1.0 wt.% EY. An EY-only emulsion was also prepared for comparison.

The emulsion formulated with EY alone underwent rapid destabilisation immediately after preparation. Complete macroscopic phase separation, accompanied by extensive droplet coalescence and oiling-off, occurred within minutes after homogenization.

This rapid failure stems from the proximity of the sample pH (3.0–3.5) to the pI of EY proteins (pH~4.5–5.0), which minimizes net surface charges, combined with a high electrolyte concentration (NaCl, potassium sorbate, organic acids) that screens electrostatic repulsion [61]. Additionally, the low EY dosage (0.9% wt.) in a dilute system (10% wt. oil) provided insufficient continuous phase viscosity or steric hindrance [62]. Consequently, the initial droplet size distribution generated during processing underwent rapid gravity-driven creaming, followed by immediate droplet coalescence due to the screened electrostatic barrier. This highlights the need to incorporate a stabilizer [63], such as CH, which improves emulsion stability not only by increasing the viscosity of the continuous phase but also by promoting the formation of a protective gel-like network in the aqueous medium. Consequently, CH acts as both a rheological modifier and a steric barrier against coalescence, a function that EY alone cannot provide under acidic conditions.

Due to its polysaccharide nature, CH typically acts as a stabilizer in O/W emulsions by forming a 3D network within the continuous phase, significantly increasing system viscosity [21]. However, the results of the present study, supported by recent literature, demonstrate that CH also plays a relevant emulsifying role. This dual functionality is attributable to its interfacial properties, which allow it to actively adsorb at the oil–water interface and provide stability beyond mere thickening of the aqueous bulk [62].

#### Evaluation of Emulsion Stability

**Figure 4:**
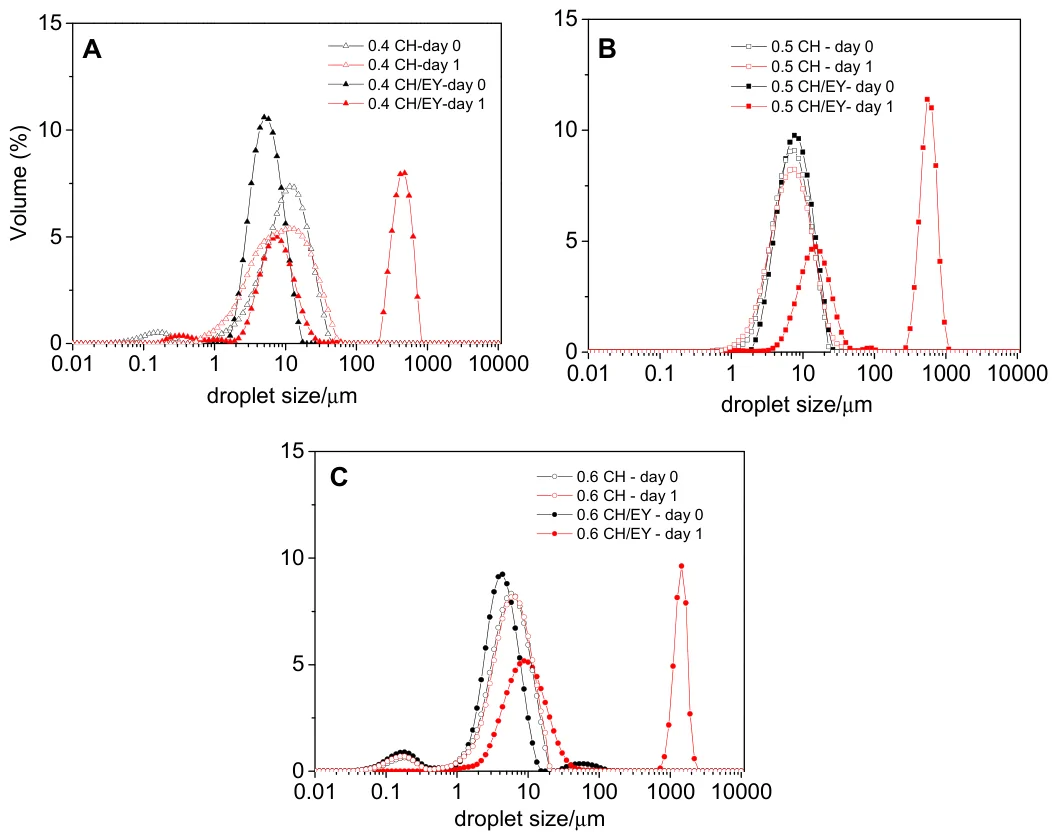
Volume droplet size distribution (%) for (**A**) chia-oil/water emulsions of CH (0.4% wt.) and CH/EY mixture (0.4, 1% wt., respectively); (**B**) CH (0.5% wt.) and CH/EY mixture (0.5, 1% wt., respectively); (**C**) CH (0.6% wt.) and CH/EY mixture (0.6, 1% wt., respectively).

The droplet size distributions (DSD) of all emulsions studied at different concentrations of CH are shown in Figure 4. Figure 4A compares the evolution of droplet size for an emulsion formulated with 0.4% wt. CH and for a mixed system containing 0.4% wt. CH and 1% wt. EY. The emulsion containing the CH/EY mixture exhibited a narrow peak centred around 5 microns, whereas the emulsion stabilized with CH alone displayed a broader distribution with a mean size around 11 microns. These results are consistent with the interfacial measurements, which indicated a higher emulsifying efficiency of EY, associated with lower interfacial tension values and enhanced interfacial viscoelastic properties.

**Figure 5:**
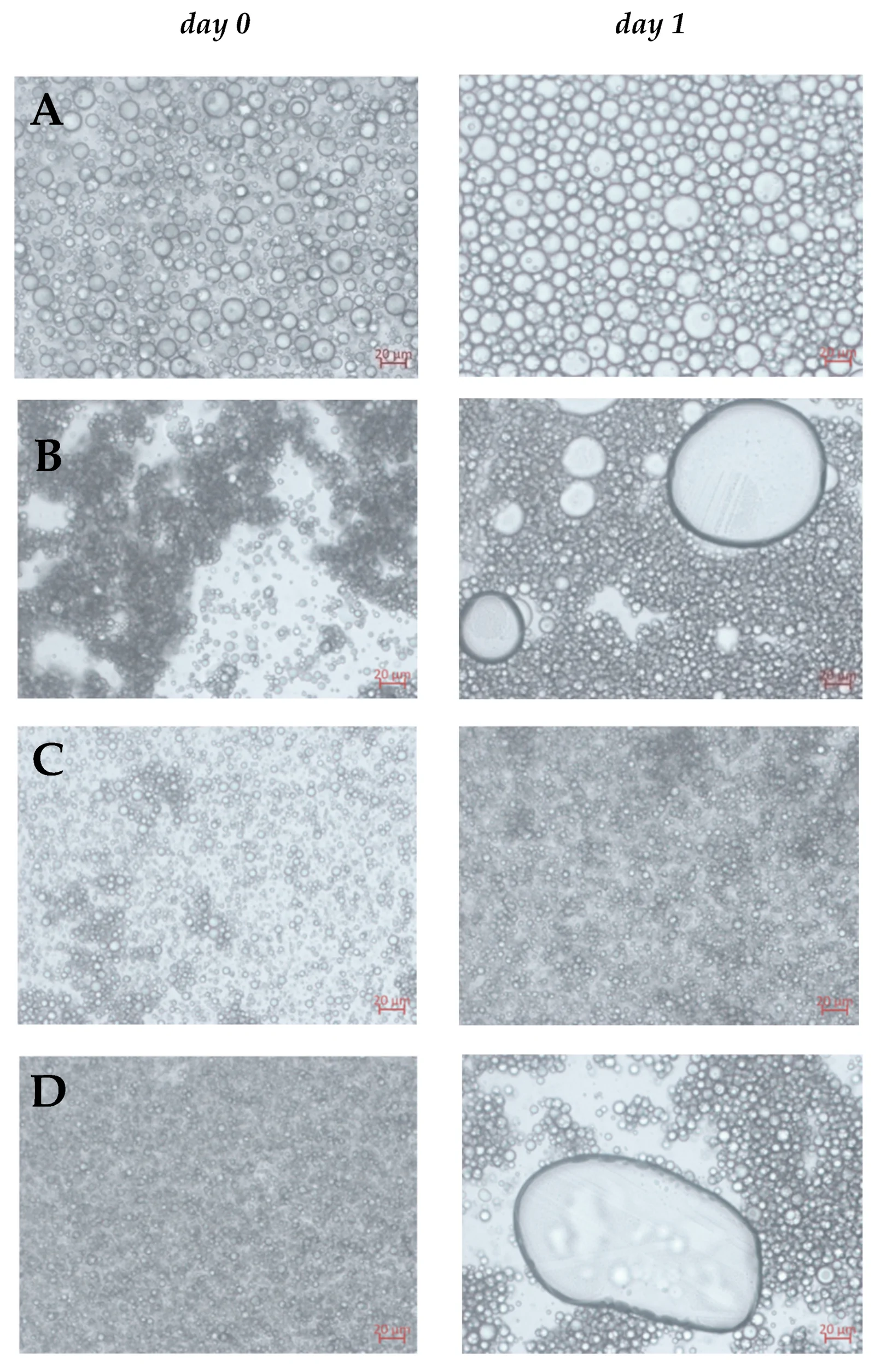
Optical micrographs of (**A**) chia oil/water emulsions containing CH (0.4 wt.%), (**B**) CH/EY (0.4 and 1 wt.%, respectively), (**C**) CH (0.6 wt.%), and (**D**) CH/EY (0.6 and 1 wt.%, respectively), obtained at day 0 and day 1. Scale bar: 20 μm.

After one day of storage, the CH/EY system exhibited a bimodal DSD, with one population slightly shifted towards larger sizes and a second population centred around 500 microns, clearly indicating the occurrence of droplet coalescence. This behaviour was corroborated by optical microscopy observations (Figure 5B). In contrast, the emulsion stabilized with CH alone maintained a single, broader distribution, indicative of slightly higher polydispersity but without evidence of significant coalescence, as also confirmed by optical micrographs (Figure 5A). This result is particularly relevant, as it reveals not only the emulsifying capacity of CH but also that CH-stabilized emulsions exhibit greater stability than those formulated with a widely used food emulsifier such as EY, despite the superior interfacial properties of the latter. This behaviour may be associated with changes in interfacial organization when CH and EY are simultaneously present. EY components are positively charged at the pH of the emulsion (approximately 3.5), since the isoelectric point of EY is around pH 4.5 [64,65]. Although interfacial measurements indicated that the interface was primarily controlled by EY molecules, electrostatic and steric incompatibilities between both biopolymers may lead to interfacial weakening [66,67], ultimately promoting droplet coalescence during storage.

Considering that the creaming process is often a precursor to droplet coalescence [66], an increase in polysaccharide concentration was evaluated as a potential stabilization strategy. As widely reported, creaming can be delayed by the action of stabilizers, which hinder droplet mobility by increasing the viscosity of the continuous phase [21]. However, increasing the CH concentration in the CH/EY mixed emulsions did not result in a significant reduction in coalescence (Figure 4B,C). In fact, at 0.6% wt. CH, a more pronounced coalescence was observed, with the second population shifting towards larger droplet sizes (approximately 1500 microns). This behaviour was also evident in optical micrographs, where droplets with sizes consistent with laser diffraction measurements were observed (Figure 5D). These results suggest that, in mixed systems, coalescence is not primarily governed by creaming but may instead be associated with changes in interfacial organization when both components are present. A similar complexity has been reported in other mixed protein–polysaccharide systems. Rodríguez Patino and Pilosof [67] demonstrated that protein–polysaccharide interactions at fluid interfaces may either improve or impair emulsion stability depending on the adsorption behaviour and structural compatibility of the biopolymers involved. Likewise, Dickinson [68] reported that competitive adsorption and interfacial incompatibility between proteins and polysaccharides can result in weaker interfacial films and enhanced susceptibility to droplet coalescence. These observations are consistent with the behaviour observed in the present study, where the incorporation of EY did not improve emulsion stability despite its superior interfacial activity, suggesting that interfacial organization rather than interfacial tension reduction alone governs the overall stability of the system.

In contrast, a clear improvement in stability was observed for emulsions stabilized exclusively with CH. At CH concentrations of 0.5% wt. and 0.6% wt., no significant changes in DSD or microstructure were detected during storage. This behaviour supports the established concept that delaying creaming through effective bulk stabilization also reduces the likelihood of droplet coalescence. Furthermore, since CH concentrations exceeded interfacial saturation in all cases, the excess polymer likely contributed to bulk structuring of the continuous phase, reinforcing the dual role of CH as both emulsifier and stabilizer.

The improved stability observed in CH-stabilized emulsions is consistent with previous studies reporting that chitosan can effectively reduce creaming and coalescence through a combination of interfacial and bulk stabilization mechanisms. For instance, Calero et al. [21] reported that increasing chitosan concentration enhanced the stability of potato-protein-stabilized emulsions by increasing the viscosity of the continuous phase and promoting the formation of a network structure capable of restricting droplet mobility. Similarly, Klinkesorn [62] described chitosan as a multifunctional biopolymer whose stabilizing effect originates not only from viscosity enhancement but also from its ability to participate in interfacial phenomena. The results obtained in the present study are in agreement with these observations, since increasing CH concentration above the interfacial saturation level significantly reduced creaming and prevented droplet coalescence during storage.

The quantitative droplet size parameters (D[3,2], D[4,3], and Span) for both single CH and mixed CH/EY systems at Day 0 and Day 1 are summarized in Table 2.

As shown in Table 2, while single CH formulations maintained low mean droplet diameters after 24 h of storage (D[4,3] < 11 μm), a pronounced destabilization was quantified for the mixed CH/EY systems. Although initial droplet sizes at Day 0 were fine and uniform (D[4,3] = 5.52–7.27 μm), D[4,3] increased dramatically by Day 1 for 0.5% and 0.6% CH/EY mixtures, reaching 276.18 ± 10.60 μm and 309.1 ± 38.92 μm, respectively (*p* < 0.05). This marked increase in volume-weighted diameter provides strong evidence of rapid droplet coalescence and phase separation in the mixed CH/EY systems, which may be associated with changes in interfacial organization arising from the simultaneous presence of both surface-active components.

**Figure 6:**
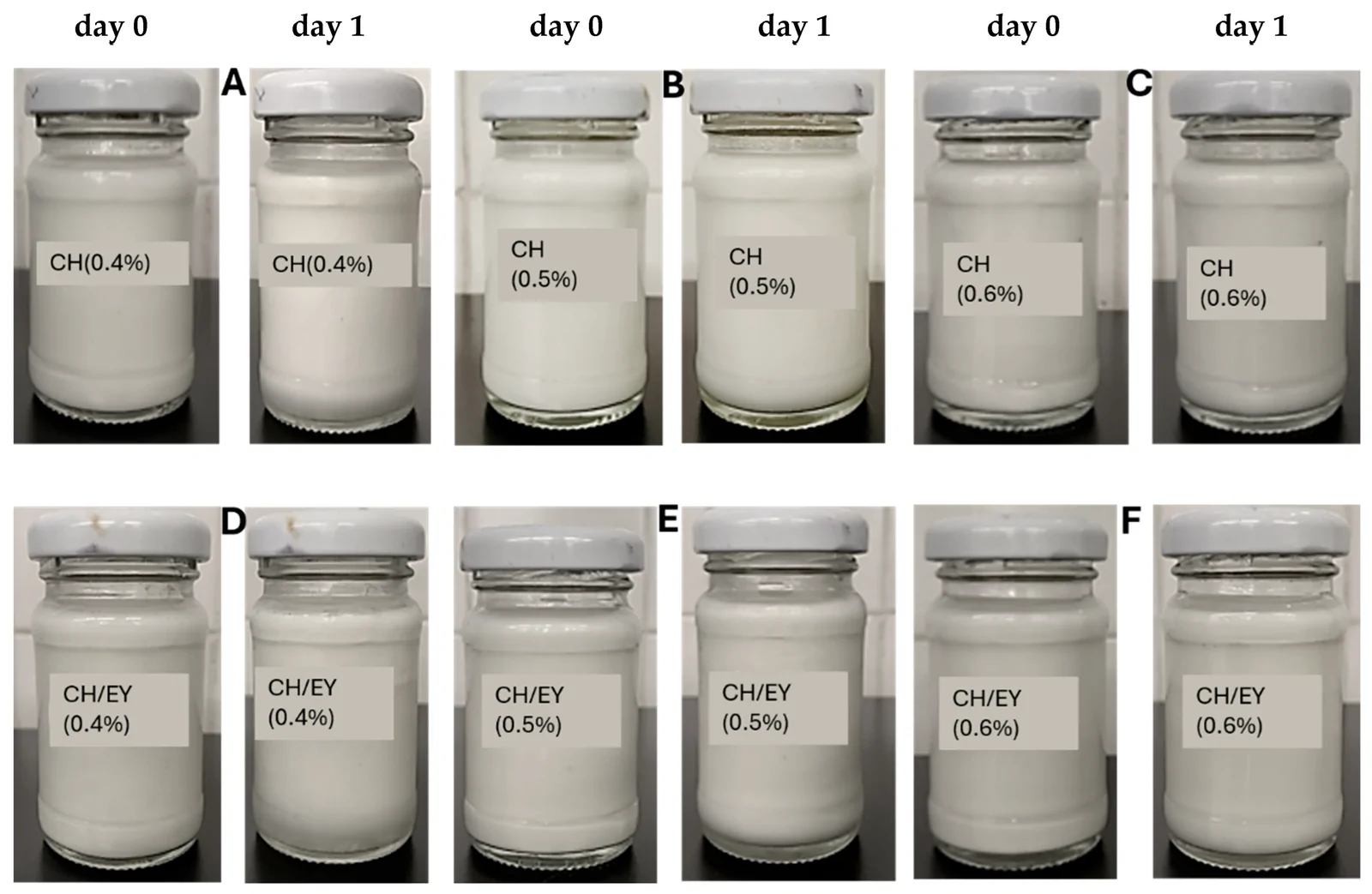
Visual appearance of (**A**) chia-oil/water emulsions of CH (0.4% wt.), (**B**) CH/EY mixture (0.4, 1% wt., respectively); (**C**) CH (0.5% wt.), (**D**) CH/EY mixture (0.5, 1% wt., respectively); (**E**) CH (0.6% wt.) and (**F**) CH/EY mixture (0.6, 1% wt., respectively) obtained at day 0 and 1.

The creaming instability of the emulsions studied could also be visually monitored at 0 and 1 day after emulsification (Figure 6A–F). At the lowest concentration CH concentration (0.4% wt., Figure 6A), creaming was clearly detected one day after emulsification by the presence of an aqueous phase layer at the bottom of the vial. In contrast, at higher CH concentration (0.5% wt. and 0.6% wt.), no visible signs of physical instability were observed (Figure 6B,C). These observations indicate that increasing CH content was sufficient to effectively delay creaming. In the case of emulsions containing the CH/EY mixture, the creaming process was evident at all CH concentration studied (Figure 6D–F). A thin aqueous layer formed at the bottom of the vial, accompanied by the upward migration of oil droplets, resulting in a more concentrated emulsion layer at the top of the vial. These macroscopic observations are consistent with the droplet size evolution previously obtained by laser diffraction.

Backscattering (BS) profiles as a function of sample height were monitored for 24 h to assess global destabilization phenomena. In general, creaming can be identified by a decrease in BS at the bottom of the vial and a corresponding increase at the top due to droplet migration, whereas a decrease in BS in the intermediate region is typically associated with droplet growth resulting from coalescence [17].

**Figure 7:**
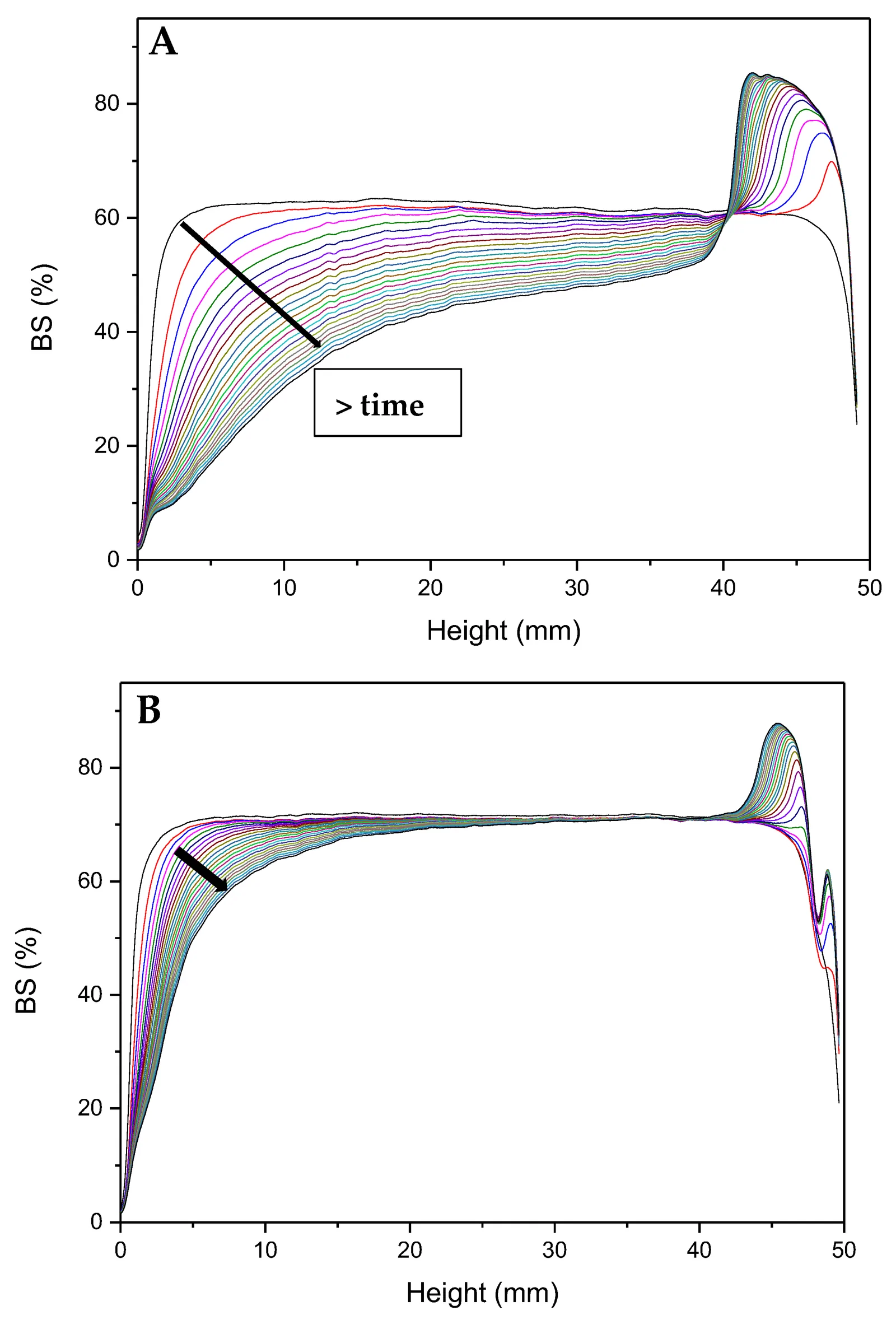

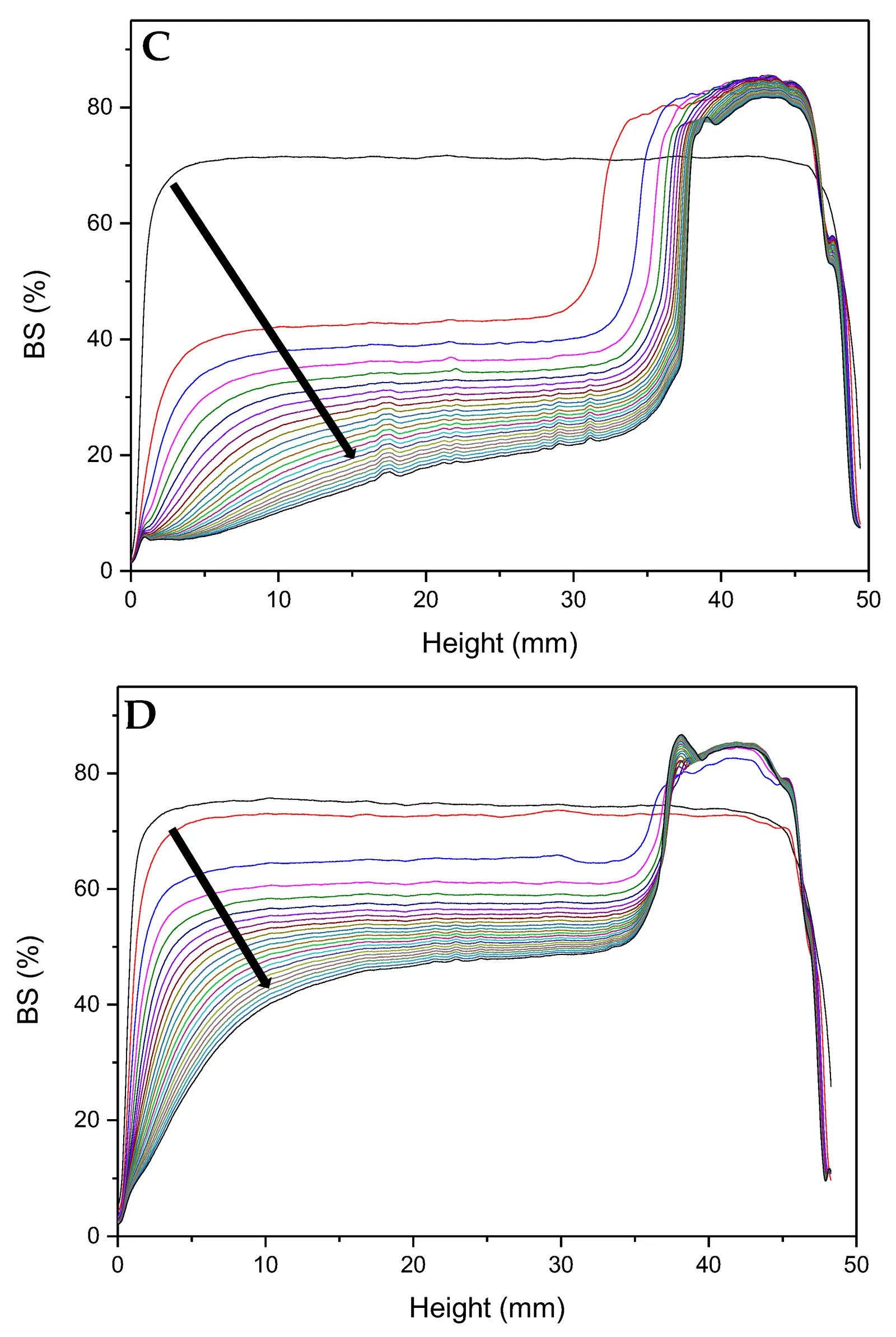
(**A**) Evolution of backscattering profiles (BS%) of (**A**) chia-O/W emulsions of CH (0.4% wt. and (**B**) 0.6% wt. and (**C**) CH/EY mixture (0.4–1% wt. and (**D**) 0.6–1% wt.) with time (24 h) as a function of the height. The arrows indicate the direction of increasing storage time.

The evolution of BS profiles for CH-stabilized emulsions at different concentrations (0.4% wt. and 0.6% wt.) is shown in Figure 7A and Figure 7B, respectively. For the most concentrated emulsion, only a slight creaming tendency was detected, characterized by a small decrease in BS at the bottom of the vial and a simultaneous increase at the top. Notably, this subtle destabilization could not be detected by the other techniques employed in this study. Moreover, no significant changes in BS were observed in the intermediate zone, indicating the absence of droplet growth due to coalescence. In contrast, the emulsion formulated with 0.4% wt. CH exhibited pronounced BS variations throughout the height of the vial, reflecting both creaming and an increase in droplet size. The improved stability observed at higher CH concentration can therefore be attributed to a significant delay in creaming and an almost complete suppression of coalescence. This behaviour supports the classical view that, in such systems, coalescence is a consequence of creaming [66,69].

The evolution of BS profiles for emulsions containing the CH/EY mixture at different CH concentrations (0.4% wt. and 0.6% wt.) is presented in Figure 7C and Figure 7D, respectively. In these systems, more pronounced BS variations were observed regardless of CH concentration. Increasing the CH content led only to a slight reduction in creaming and coalescence.

These results highlight a fundamental and often overlooked challenge in complex food formulation: combining an emulsifier with a stabilizer is by no means a simple or additive process. In single systems, egg yolk provides effective interfacial coverage, yet its inability to impart continuous-phase viscosity leaves the emulsion highly vulnerable to rapid creaming. Introducing chitosan as a bulk thickener might intuitively seem like the ideal strategy to overcome this limitation; however, because chitosan is not a passive polymer but a surface-active agent itself, this combination creates severe interfacial conflict. Rather than improving emulsion stability, the simultaneous presence of egg yolk and chitosan resulted in poorer stability than that observed for CH alone, suggesting that changes in interfacial organization may occur when both surface-active components are present.

This behaviour is consistent with droplet coalescence acting as a primary destabilisation mechanism in mixed systems and may be associated with changes in interfacial organization when both components are present [70]. This interfacial failure triggers immediate droplet growth in the bulk phase; thus, rather than viewing creaming as a precursor to coalescence, as is typical in conventional systems, these results support the hypothesis that early interfacial failure and droplet growth accelerate gravitational separation according to Stokes’ Law, thereby strongly dictating the observed creaming kinetics in CH/EY emulsions.

**Figure 8:**
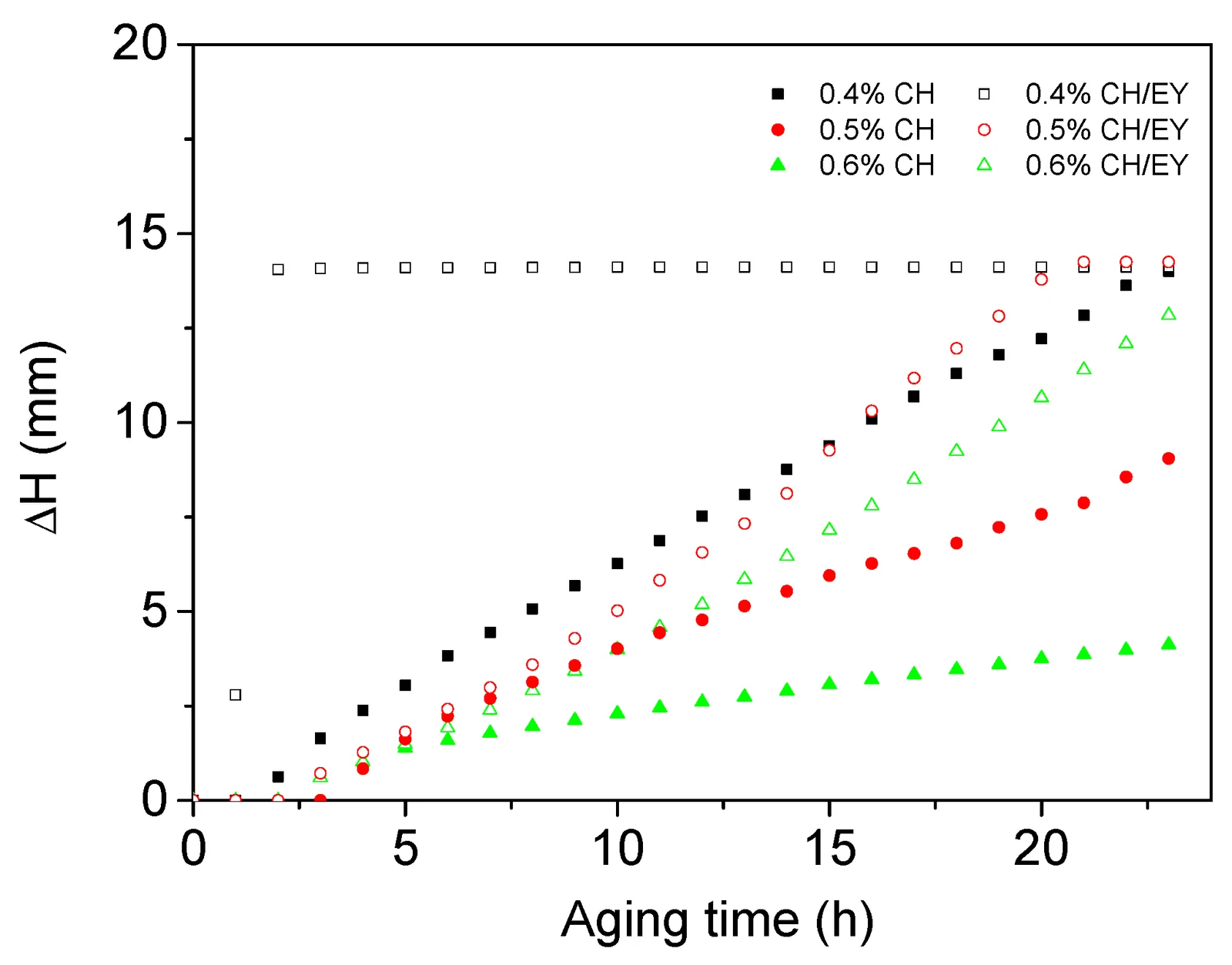
Evolution of creaming with time for the different chia O/W emulsions analysed, where ΔH represents the change in creaming layer height (mm).

Finally, Figure 8 shows the creaming rate for all the emulsions analysed. The addition of CH provoked a clear reduction in the creaming rate for both systems, with a more pronounced effect observed for emulsions stabilised exclusively with CH. An increase in CH concentration led to up to a 4.2-fold reduction in the creaming rate compared to the EY-only control emulsion. This marked stabilization highlights the bifunctional role of chitosan: besides contributing to interfacial organization at the droplet surface, CH progressively structures the continuous phase by increasing bulk viscosity, thereby retarding gravitational separation [1].

## 4. Conclusions

CH stands out as a highly promising biopolymer for food applications due to its dual functionality as both a natural stabilizer and emulsifier under acidic food-emulsion conditions. From a commercial perspective, its ability to replace synthetic additives in food emulsion models makes it a promising candidate for developing clean-label products with improved physical stability.

Although EY exhibited superior interfacial activity, CH-stabilized systems showed significantly enhanced macroscopic stability against both creaming and coalescence. Interfacial characterization demonstrated that EY dominated adsorption and controlled the rheological properties of the interfacial film, whereas CH reached the interface rapidly but formed weaker interfacial networks. Despite this, emulsions stabilized exclusively with CH displayed greater physical stability, highlighting the key role of bulk structuring effects.

In mixed CH/EY systems, the simultaneous presence of both components resulted in greater destabilization than that observed for CH-only emulsions, suggesting that changes in interfacial organization may occur when both surface-active components are present. In contrast, increasing CH content in single-biopolymer emulsions delayed creaming and practically suppressed coalescence, resulting in a 4.2-fold reduction in creaming rate relative to the EY control. These findings highlight the complexity of combining surface-active components in food emulsions and demonstrate that the incorporation of EY did not provide additional stabilization under the conditions investigated.

This work serves as the necessary starting point of a broader, ongoing research initiative. Demonstrating this baseline performance allows us to target future chemical modifications of chitosan (e.g., amphiphilic derivatization), optimizing its interfacial capacity to outperform classic emulsifiers even under harsh real-world conditions. Future research could also include advanced microscopy techniques, such as scanning electron microscopy (SEM), cryo-scanning electron microscopy (cryo-SEM) or confocal laser scanning microscopy (CLSM), to directly visualize microstructural organization and provide complementary evidence of the mechanisms proposed in this study.

## Figures and Tables

**Table 1:** Aqueous phase composition. The components are listed in order of addition. X value of CH indicates that the amount of CH was used (0.4, 0.5 and 0.6%).

Aqueous Phase Composition (%, wt.)	
CH/EY	CH
CH (X %)	CH (X %)
Milli-Q water (94.55—X %)	Milli-Q water (95.45—X %)
Vinegar (3.5%)	Vinegar (3.5%)
EY (1%)	--
Potassium sorbate (0.1%)	Potassium sorbate (0.1%)
Salt (0.65%)	Salt (0.75%)
Lemon juice (0.2%)	Lemon juice (0.2%)

**Table 2:** Quantitative droplet size parameters (D[3,2], D[4,3], and Span) for emulsions prepared with chitosan alone (CH) and chitosan/egg yolk mixtures (CH/EY) at day 0 and day 1.

Sample	*D*[3,2]	*D*[4,3]	Span
0.4 CH—day 0	5.06 ± 0.14 ^a^	7.44 ± 0.22 ^A^	1.60 ± 0.05 ^α^
0.5 CH—day 0	6.27 ± 0.10 ^b^	8.15 ± 0.16 ^A^	2.89 ± 0.01 ^β^
0.6 CH—day 0	1.10 ± 0.05 ^c^	3.99 ± 0.01 ^B^	1.65 ± 0.02 ^α^
0.4 CH—day 1	4.48 ± 0.03 ^d^	10.89 ± 0.10 ^C^	2.89 ± 0.02 ^β^
0.5 CH—day 1	4.96 ± 0.49 ^a^	7.88 ± 1.48 ^A^	1.69 ± 0.22 ^α^
0.6 CH—day 1	4.66 ± 0.01 ^a^	7.55 ± 0.02 ^A^	1.80 ± 0.01 ^α^
0.4 CH/EY—day 0	4.44 ± 0.02 ^a,^*	5.52 ± 0.03 ^A,^*	1.33 ± 0.01 ^α,^*
0.5 CH/EY—day 0	5.28 ± 0.05 ^b,^*	7.27 ± 0.01 ^B,^*	1.57 ± 0.01 ^α,^*
0.6 CH/EY—day 0	1.37 ± 0.01 ^c,^*	5.83 ± 0.02 ^A,^*	1.85 ± 0.01 ^α,^*
0.4 CH/EY—day 1	2.52 ± 0.06 ^d,^*	150.91 ± 0.96 ^C,^*	1.92 ± 0.16 ^α,^*
0.5 CH/EY—day 1	14.15 ± 1.05 ^e,^*	276.18 ± 10.60 ^D,^*	1.74 ± 0.27 ^α,^*
0.6 CH/EY—day 1	23.35 ± 2.90 ^f,^*	309.14 ± 38.92 ^D,^*	1.50 ± 0.02 ^α,^*

Values are presented as mean ± standard deviation (n = 3). Statistical analysis was performed separately for single-biopolymer systems (CH) and mixed systems (CH/EY, denoted with an Asterisk. Different letters indicate statistically significant differences (*p* < 0.05).

## Data Availability

The original contributions presented in this study are included in the article. Further inquiries can be directed to the corresponding author.
